# Gestational age at birth and the risk of disability pension in early adulthood: a whole-population register study of people born in Finland in 1987–90

**DOI:** 10.1093/ije/dyag127

**Published:** 2026-07-29

**Authors:** Oksanen Päivi, Tikanmäki Marjaana, Alenius Suvi, Metsälä Johanna, Gissler Mika, Lahti-Pulkkinen Marius, Heikkilä Katriina, Räikkönen Katri, Ala-Mursula Leena, Kajantie Eero

**Affiliations:** Clinical Medicine Research Unit, Medical Research Center, Oulu University Hospital, University of Oulu, Oulu, Finland; Public Health Unit, Finnish Institute for Health and Welfare, Helsinki and Oulu, Finland; Clinical Medicine Research Unit, Medical Research Center, Oulu University Hospital, University of Oulu, Oulu, Finland; Public Health Unit, Finnish Institute for Health and Welfare, Helsinki and Oulu, Finland; Clinical Medicine Research Unit, Medical Research Center, Oulu University Hospital, University of Oulu, Oulu, Finland; Public Health Unit, Finnish Institute for Health and Welfare, Helsinki and Oulu, Finland; Children’s Hospital, University of Helsinki and Helsinki University Hospital, Helsinki, Finland; Clinical Medicine Research Unit, Medical Research Center, Oulu University Hospital, University of Oulu, Oulu, Finland; Public Health Unit, Finnish Institute for Health and Welfare, Helsinki and Oulu, Finland; Public Health Unit, Finnish Institute for Health and Welfare, Helsinki and Oulu, Finland; Region Stockholm, Academic Primary Health Care Centre, Stockholm, Sweden; Department of Molecular Medicine and Surgery, Karolinska Institutet, Stockholm, Sweden; Research Centre for Child Psychiatry and Invest Research Flagship, University of Turku, Turku, Finland; Department of Psychology, University of Helsinki, Helsinki, Finland; Clinical Medicine Research Unit, Medical Research Center, Oulu University Hospital, University of Oulu, Oulu, Finland; Department of Psychology, University of Helsinki, Helsinki, Finland; Department of Obstetrics and Gynaecology, Helsinki University Hospital, Helsinki, Finland; Research Unit of Population Health, University of Oulu, Oulu, Finland; Wellbeing Services County of North Ostrobothnia Pohde, Oulu, Finland; Clinical Medicine Research Unit, Medical Research Center, Oulu University Hospital, University of Oulu, Oulu, Finland; Public Health Unit, Finnish Institute for Health and Welfare, Helsinki and Oulu, Finland; Department of Clinical and Molecular Medicine, Norwegian University of Science and Technology, Trondheim, Norway; Public Health, Folkhälsan Research Center, Helsinki, Finland

**Keywords:** disability pension, early-term, post-term, preterm

## Abstract

**Background:**

Preterm (<37 weeks), early-term (37–38 weeks), or post-term (42 weeks) birth may elevate the risk of mental, neurodevelopmental, and cardiopulmonary disease in adulthood, potentially impacting work capacity.

**Methods:**

We examined the association between gestational age and early disability pension (DP) among 228 572 Finnish individuals born between 1 January 1987 and 30 September 1990. We identified the first DP decision from ages 16 to ∼32 years (2003–20) by using linked birth and DP records, including timing and diagnosis. Adjusted hazard ratios (HRs) with 95% confidence intervals were estimated by using Cox regression, with full-term birth (39–41 weeks) as the reference.

**Results:**

A total of 9224 individuals (4.0%) received DPs. Adjusted for sex and birth year, the HRs for DP by gestational age groups of 23–31, 32–33, 34–36, 37–38, and 42 weeks were 5.20 (4.52–5.99), 2.47 (2.04–3.00), 1.55 (1.41–1.70), 1.22 (1.16–1.29), and 1.12 (1.01–1.25). Among preterm adults, the most common diagnoses included cerebral palsy, intellectual disability, depression, and schizophrenia. For early-term and post-term adults, depression and schizophrenia were the most frequent.

**Conclusion:**

Preterm, early-term, and post-term births were associated with early DPs, with slightly different diagnostic profiles. The risk was highest among those born very preterm (<32 weeks). High-quality perinatological care is crucial regarding future careers and societal costs.

Key Messages

- We examined whether being born before or after the gestational age of 39–41 weeks is associated with an increased risk of early adulthood disability pension (DP), particularly due to mental and neurodevelopmental disorders, and whether this association is stronger among individuals born small for gestational age or from low socio-economic backgrounds, or varies by sex.
- We found that DPs in young adulthood were mainly granted due to mental, behavioural, and neurodevelopmental disorders, with preterm birth associated with a 1.6- to 5.2-fold increased risk in a dose–response manner, and elevated risks were also observed for early-term (1.2-fold) and post-term (1.1-fold) births compared with full-term birth.
- The elevated risk of DP among those born late-preterm and early-term highlights a public health concern due to the size of these groups, emphasizing the importance of high-quality perinatal care, sustained workforce participation, and proactive, tailored rehabilitation to prevent early retirement and reduce societal costs.

## Introduction

Preterm birth (<37 completed weeks of gestation) is linked to various health challenges, especially among those born earliest in gestation, including intellectual disability [1], cerebral palsy (CP) [2], visual impairment [3], mental disorders [4, 5], and increased cardiometabolic risk [6, 7]. Emerging evidence also indicates that early-term birth (37–38 weeks) is associated with these increased health risks [8]. Post-term birth (≥42 weeks) may elevate the risk of obesity [9, 10], mental disorders [11], and behavioural and emotional problems [12]. These disadvantages may contribute to lower educational attainment [13–16], work capacity, and income [15, 16] in adulthood.

Previous studies have linked preterm birth with a higher risk of requiring a disability pension (DP) [15–17] in adulthood. In Norway [16], a DP prevalence of 8.7% was observed among those born at 30 weeks 6 days or less versus 1.7% among full-term (>37 weeks) individuals at ages 20–36 years. In Sweden, the need for disability support (sickness pension, disability allowance, and/or disability assistance) among 23- to 29‑year‑olds [15] became more common with decreasing gestational age (GA). In Denmark, birth before 39 weeks was associated with higher odds of receiving a DP at age 28 years [17].

Previous studies, mostly based on cohorts born in around the 1970s when prenatal and neonatal care was less advanced, have not comprehensively covered the full GA range (including post-term), tracked DPs from as early as age 16 years, or examined the diagnostic causes of the need for DPs. Our study addresses these gaps by using nationwide register data on individuals born in Finland between 1987 and 1990, covering the full GA range up to 42 weeks. We conducted a 16-year follow-up from age 16 years (2003–20), enabling survival analyses, diagnostic profiling, and identification of those who transitioned directly to DPs at age 16 years—the earliest eligible age in Finland—allowing separate analysis of later transitions. Our primary hypothesis was that non-optimal GA increases the DP risk, particularly due to mental and neurodevelopmental disorders [5, 18]. We also hypothesized that the association of GA with DP may be stronger among those born as small for GA (SGA) [11] or among families of low socio-economic position (SEP) [19] and it may vary according to sex [16, 20].

## Material and methods

### Data sources and study population

Individuals born from 1 January 1987 to 30 September 1990 were identified from the Finnish Medical Birth Register and Central Population Register, and information on deaths and emigrations (2020) was obtained from Statistics Finland. GA was retrieved from the Finnish Medical Birth Register, based on ultrasound before 20 gestational weeks or last menstrual period. DP data (for years 2006–20) were obtained from the Finnish Centre for Pensions (FCP) and the Social Insurance Institution of Finland (SII, years 2003–19). All datasets were linked by using encrypted personal identifiers within Statistics Finland’s secure remote access system, FIONA [21].

**Figure 1:**
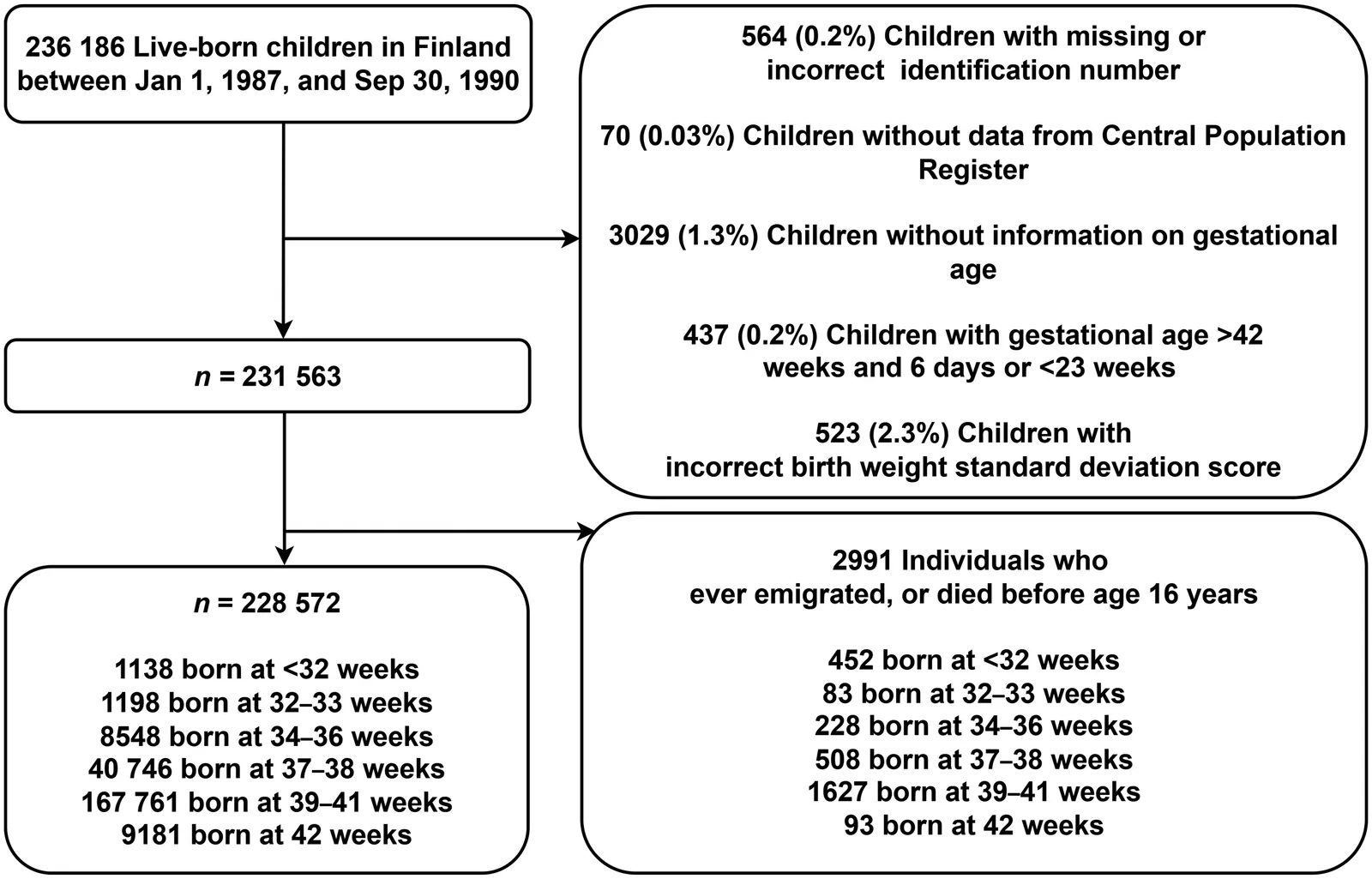
Flowchart of the study.

Of the 236 186 live-born children in Finland during the study period, 4623 (1.96%) were excluded due to missing or incorrect identification numbers, birthweight SD scores, or GAs. After the exclusion of individuals who emigrated or died before age 16 years (*n *= 2991; 1.3%), the remaining 228 572 (97.0%) participants were categorized into six GA groups: very preterm (23–31 weeks, 0.5%), moderately preterm (32–33 weeks, 0.5%), late preterm (34–36 weeks, 3.7%), early-term (37–38 weeks, 17.8%), full-term (39–41 weeks, reference, 73.4%), and post-term (42 weeks, 4.0%) (Table 1 and Fig. 1).

**Table 1:** Characteristics at peri- and neonatal age, childhood, and adulthood by GA compared with the reference group (individuals born at 39 + 0–41 + 6 weeks of gestation) from the Finnish 1987–90 Birth Cohort study (*n *= 228 572).

Variable	<32 + 0 weeks of gestation (*n *= 1138)			*P* value^a^	32 + 0–33 + 6 weeks of gestation (*n *= 1198)			*P* value^a^	34 + 0–36 + 6 weeks of gestation (*n *= 8548)			*P* value^a^	37 + 0–38 + 6 weeks of gestation (*n *= 40 746)			*P* value^a^	39 + 0–41 + 6 weeks of gestation (reference) (*n *= 167 761)			42–42 + 6 weeks of gestation (*n *= 9182)			*P* value^a^
	*n*	%	Mean (SD)		*n*	%	Mean (SD)		*n*	%	Mean (SD)		*n*	%	Mean (SD)		*n*	%	Mean (SD)	*n*	%	Mean (SD)	
Men	651	57.2			657	54.8			4603	53.8			21 474	52.7			84 849	50.6		4732	51.5		
Birth year			1988.4 (1.1)	1			1988.5 (1.1)	.574			1988.4 (1.1)	.947			1988.4 (1.1)	1			1988.4 (1.1)			1988.5 (1.1)	<.001
1987	297	26.1			290	24.2			2152	25.2			10 080	24.7			42 819	25.5		2203	24.0		
1988	281	24.7			317	26.5			2257	26.4			11 370	27.9			44 726	26.7		2209	24.1		
1989	319	28.0			318	26.5			2332	27.3			10 827	26.6			44 691	26.6		2625	28.6		
1990 (January–September)	241	21.2			273	22.8			1807	21.1			8469	20.8			35 525	21.2		2144	23.4		
**Peri- and neonatal**																							
Age of the mother at birth			29.2 (5.7)	.001			29.1 (5.9)	.001			28.8 (5.6)	<.001			28.9 (5.4)	<.001			28.4 (5.2)			27.8 (4.9)	<.001
Parity (at least one partus before this pregnancy)	562	49.4			600	50.1			3894	45.6			15 472	38.0			65 577	39.1		4849	52.8		
Maternal hypertension	211	18.5			257	21.5			1186	13.9			3821	9.4			8191	4.9		300	3.3		
Maternal gestational diabetes	30	2.6			47	3.9			411	4.8			2062	5.1			4588	2.7		113	1.2		
Maternal smoking																							
No maternal smoking	898	78.9			910	76.0			6810	79.7			33 568	82.4			140 256	83.6		7537	82.1		
Maternal smoking during pregnancy, <10 cigarettes/day	109	9.6			114	9.5			720	8.4			3272	8.0			12 770	7.6		788	8.6		
Maternal smoking during pregnancy, >10 cigarettes/day	107	9.4			137	11.4			846	9.9			3126	7.7			11 527	6.9		703	7.7		
Unknown	24	2.1			37	3.1			172	2.0			780	1.9			3208	1.9		153	1.7		
GA (weeks)			29.5 (2.0)	<.001			33.1 (0.6)	<.001			35.9 (0.8)	<.001			38.2 (0.5)	<.001			40.3 (0.8)			42.2 (0.2)	<.001
Birthweight (g)			1329 (366)	<.001			1976 (391)	<.001			2697 (481)	<.001			3321 (480)	<.001			3681 (460)			3859 (463)	<.001
Birthweight SD score			−0.08 (1.47)	.007			−0.31 (1.48)	<.001			−0.18 (1.30)	<.001			−0.01 (1.15)	<.001			0.02 (1.02)			−0.03 (1.01)	<.001
SGA	130	11.4			169	14.1			723	8.5			1512	3.7			3266	1.9		202	2.2		
Appropriate for gestational age	937	82.3			974	81.3			7443	87.1			37 513	92.1			15 9261	94.9		8731	95.1		
Large for gestational age	71	6.2			55	4.6			382	4.5			1721	4.2			5234	3.1		248	2.7		
**Childhood**																							
Parental educational level, highest ever attained																							
Basic or unknown	84	7.4			118	9.8			670	7.8			2690	6.6			8878	5.3		525	5.7		
Secondary, less than tertiary	771	67.8			775	64.7			5668	66.3			26 445	64.9			110 192	65.7		6042	65.8		
Tertiary or more	283	24.9			305	25.5			2210	25.9			11 611	28.5			48 691	29.0		2614	28.5		
Parental SEP																							
Other^b^ or missing information	56	4.9			54	4.5			425	5.0			1786	4.4			6400	3.8		300	3.3		
Manual workers	169	14.9			189	15.8			1234	14.4			5481	13.5			20 312	12.1		1098	12.0		
Lower white-collar	473	41.6			492	41.1			3537	41.4			16 380	40.2			68 293	40.7		3760	41.0		
Higher white-collar	440	38.7			463	38.6			3352	39.2			17 099	42.0			72 756	43.4		4023	43.8		
**Follow-up during the years 2003–20 (at age 16–33 years)**																							
No disability pension	940	82.6			1092	91.2			8064	94.3			38 908	95.5			16 1523	96.3		8799	95.8		
Emigrated, no DP^c^	25	2.2			29	2.4			213	2.5			1127	2.8			5023	3.0		258	2.8		
Died (no DP)	12	1.1			14	1.2			62	.7			327	.8			1214	.7		68	.7		
DP during follow-up time	198	17.4			106	8.8			484	5.7			1834	4.5			6222	3.7		380	4.1		
Died after receiving DP	9	.8			5	.4			28	.3			67	.2			258	.2		15	.2		

a The *P* values refer to two-sided tests for differences compared with those born at 39 + 0–41 + 6 weeks of gestation (two-way analysis of variance).

b Group ‘Other’ includes individuals not engaged in any occupation, those in military or civil service, family members without their own occupation, persons living on property income or savings, institutional inmates, or cases for which socio-economic status cannot be determined. This group also includes students, retired persons, and long-term unemployed.

c Emigrated during the follow-up period, did not have a DP before emigration or after returning.

*P* values regarding categorical analyses from Pearson’s χ2 test for differences compared with those born at 39 + 0–41 + 6 weeks of gestation were <.05 except for ‘Died (no DP)’.

No missing data except ‘Age of the mother at birth’, for which data were missing for one individual in the reference group born at 39 + 0–41 + 6 weeks of gestation.

### Covariates

Covariates were selected based on prior literature linking them to preterm birth and DP [15, 16, 22, 23], considering gendered labour-market opportunities, work disability trends, sex-specific diagnostic patterns [24–26], and group differences, as shown in Table 1.

We ran two sets of models that were built on each other. Model 1 included sex (male vs. female) and year of birth (1990 as reference). In Model 2, we additionally included previously described [22] covariates: parity (at least one live birth before this pregnancy, yes vs. no), singleton pregnancy (yes vs. no), gestational hypertension (no vs. yes), gestational diabetes (no vs. yes), maternal smoking during pregnancy (no vs. <10 cigarettes/day, ≥10 cigarettes/day, missing information), parental highest-attained educational level (basic or unknown, secondary, tertiary or more), and parental highest-attained SEP during the years 1995–2014 (higher white-collar, lower white-collar, manual workers, and other/missing information). The ‘Other’ group included conscripts, individuals without an occupation or living on property income or savings, institutionalized individuals, students, retirees, or the long‑term unemployed. Additionally, Model 2 included maternal age at birth and the index individual’s birthweight SD score based on the national growth charts [27] as continuous covariates. Birthweight SD scores were also categorized as: SGA defined as birthweight >2 SD below the population mean, appropriate for GA (AGA) as within +/−2 SDs around the mean, and large for GA (LGA) as >2 SD above the mean [28, 29].

### Outcomes

Primary outcomes included the timing and occurrence of the first DP decision, along with ICD-10-based (International Statistical Classification of Diseases and Related Health Problems 10th Revision) medical diagnoses obtained from SII and FCP. Diagnoses were analysed at the ICD-10 three-digit level (e.g. F00–F99), including all related four-character subcategories (Tables 2 and 3).

**Table 2:** Risk of DP and related key diagnoses by GA compared with the reference group (individuals born at 39 + 0–41 + 6 weeks of gestation) from the Finnish 1987–90 Birth Cohort study (*n *= 228 572).

Weeks of gestation (*N*)		<32 + 0 (*n *= 1138)				32 + 0–33 + 6 (*n *= 1198)				34–36 + 6 (*n *= 8548)				37–38 + 6 (*n *= 40 746)				39–41 + 6 (*n *= 167 761)		42–42 + 6 (*n *= 9182)			
	M	*n*	%	HR (95% CI)	*P* value^a^	*n*	%	HR (95% CI)	*P* value^a^	*n*	%	HR (95% CI)	*P* value^a^	*n*	%	HR (95% CI)	*P* value^a^	*n*	%	*n*	%	HR (95% CI)	*P* value^a^
No DP	1	940	82.6	0.97 (0.91–1.04)	.38	1092	91.2	0.95 (0.89–1.01)	.085	8064	94.34	0.99 (0.97–1.02)	.53	38 908	95.5	0.99 (0.98–1.00)	.022	161 523	96.3	8799	95.83	1.02 (1.00–1.05)	.042
	2			0.98 (0.92–1.05)	.53			0.96 (0.90–1.02)	.143			1.00 (0.98–1.02)	.817			0.99 (0.98–1.00)	.034					1.02 (1.00–1.05)	.041
All DP causes together	1	198	17.4	5.20 (4.52–5.99)	<.001	106	8.8	2.47 (2.04–3.00)	<.001	484	5.66	1.55 (1.41–1.70)	<.001	1834	4.5	1.22 (1.16–1.29)	<.001	6222	3.7	380	4.14	1.12 (1.01–1.25)	.028
	2			4.92 (4.26–5.69)	<.001			2.24 (1.84–2.72)	<.001			1.45 (1.32–1.59)	<.001			1.18 (1.12–1.25)	<.001					1.13 (1.02–1.25)	.022
ICD-10 F00–F99	1	109	9.6	3.64 (3.01–4.40)	<.001	76	6.3	2.25 (1.80–2.82)	<.001	344	4.02	1.40 (1.25–1.56)	<.001	1406	3.5	1.19 (1.12–1.26)	<.001	4912	2.9	320	3.49	1.20 (1.07–1.34)	.002
	2			3.39 (2.80–4.12)	<.001			2.01 (1.60–2.53)	<.001			1.29 (1.16–1.45)	<.001			1.15 (1.08–1.22)	<.001					1.20 (1.08–1.35)	.001
ICD-10 F20–F29	1	19	1.7	0.83 (0.53–1.31)	.42	15	1.3	0.93 (0.56–1.55)	.784	85	.99	1.02 (0.82–1.28)	.829	325	.8	0.89 (0.79–1.01)	.071	1289	.8	88	.96	1.15 (0.93–1.43)	.206
	2			0.77 (0.48–1.21)	.26			0.86 (0.51–1.44)	.56			0.98 (0.78–1.24)	.883			0.87 (0.77–0.99)	.031					1.15 (0.93–1.43)	.196
ICD-10 F30–F39	1	24	2.1	0.82 (0.55–1.23)	.34	17	1.4	0.77 (0.48–1.25)	.291	102	1.19	0.93 (0.76–1.14)	.485	512	1.3	1.05 (0.95–1.16)	.325	1770	1.1	96	1.05	0.89 (0.73–1.09)	.269
	2			0.78 (0.52–1.18)	.24			0.76 (0.47–1.23)	.263			0.91 (0.73–1.12)	.35			1.04 (0.94–1.15)	.466					0.88 (0.72–1.08)	.233
ICD-10 F40–F48	1	7	0.6	0.91 (0.43–1.93)	.81	12	1.0	2.23 (1.26–3.96)	.006	27	0.32	0.98 (0.67–1.45)	.932	104	0.3	0.86 (0.70–1.07)	.175	436	0.3	25	0.27	0.95 (0.64–1.43)	.819
	2			0.91 (0.43–1.93)	.81			2.26 (1.24–4.11)	.007			0.98 (0.65–1.47)	.928			0.86 (0.69–1.07)	.175					0.94 (0.63–1.41)	.755
ICD-10 F70–F79	1	43	3.8	2.14 (1.57–2.91)	<.001	23	1.9	1.88 (1.25–2.85)	.003	81	0.95	1.38 (1.10–1.74)	.005	296	0.7	1.38 (1.10–1.74)	.001	836	0.5	65	0.71	1.28 (1.00–1.65)	.055
	2			2.04 (1.49–2.80)	<.001			1.69 (1.10–2.59)	.016			1.20 (0.95–1.52)	.124			1.20 (1.05–1.37)	.007					1.31 (1.02–1.68)	.038
ICD-10 F80–F89	1	8	0.7	1.17 (0.58–2.36)	.66	7	0.6	1.53 (0.73–3.24)	.263	24	.28	1.03 (0.68–1.55)	.905	97	0.2	0.97 (0.78–1.22)	.801	354	0.2	25	0.27	1.17 (0.78–1.76)	.442
	2			1.07 (0.52–2.19)	.85			1.31 (0.60–2.86)	.492			0.95 (0.62–1.45)	.81			0.95 (0.76–1.19)	.652					1.16 (0.77–1.75)	.469
ICD-10 G80	1	58	5.1	65.4 (43.5–98.4)	<.001	16	1.3	28.3 (15.8–50.7)	<.001	13	0.15	4.83 (2.58–9.04)	<.001	15	0.0	1.37 (0.76–2.49)	.296	39	0.0	NR			
	2			72.2 (46.8–111.2)	<.001			32.6 (17.7–60.0)	<.001			5.02 (2.65–9.51)	<.001			1.41 (0.78–2.56)	.258						

CI, confidence interval; HR, hazard ratio; M, Model; NR, data not reported when the number of observations was up to three, to ensure individual privacy; F00–F99, Mental, behavioural, and neurodevelopmental disorders; F20–F29, Schizophrenia, schizotypal, delusional, and other non-mood psychotic disorders; F30–F39, Mood [affective] disorders; F40–F48, Anxiety, dissociative, stress-related, somatoform, and other non-psychotic mental disorders; F70–F79, Intellectual disabilities; F80–F89, Pervasive and specific developmental disorders; G80, cerebral palsy.

Model 1: Sex, Year of birth. Model 2: Sex, Year of birth, Age of the mother at birth, Parity (at least one partus before this pregnancy) (yes vs. no), Singleton pregnancy (yes vs. no), Maternal hypertension (yes vs. no), Maternal gestational diabetes (yes vs. no), Maternal smoking during pregnancy, <10 cigarettes/day (yes vs. no), Maternal smoking during pregnancy, >10 cigarettes/day (yes vs. no), Maternal smoking during pregnancy, Information missing (yes vs. no), Birthweight SD score, Parental highest ever attained SEP: Lower white-collar (yes vs. no), Manual workers (yes vs. no), Other (yes vs. no; includes individuals not engaged in any occupation, those in military or civil service, family members without their own occupation, persons living on property income or savings, institutional inmates, students, retired persons, long-term unemployed, or cases for which socio-economic status cannot be determined), Missing information (yes vs. no), Parental educational level, highest ever attained: Basic or unknown, Secondary, less than tertiary, and tertiary or more.

a The *P* values refer to the adjusted hazard ratios (HRs) with 95% confidence intervals (CIs) estimated using Cox regression, with those born at 39 + 0–41 + 6 weeks of gestation as the reference.

**Table 3:** DP frequencies of key diagnoses by GA compared with the reference group (individuals born at 39 + 0–41 + 6 weeks of gestation) from the Finnish 1987–90 Birth Cohort study (*n *= 228 572).

Weeks of gestation (*N*)	<32 + 0 (*n *= 1138)		32 + 0–33 + 6 (*n *= 1198)		34–36 + 6 (*n *= 8548)		37–38 + 6 (*n *= 40 746)		39–41 + 6 (*n *= 167 761)		42–42 + 6 (*n *= 9182)		
ICD-10 group	*n*	%	*n*	%	*n*	%	*n*	%	*n*	%	*n*	%	*P* value^a^
ICD-10 F00–F99	109	9.6	76	6.3	344	4.0	1406	3.5	4912	2.9	320	3.5	<.001
ICD-10 F20–F29	19	1.7	15	1.3	85	1.0	325	0.8	1289	0.8	88	1.0	<.001
ICD-10 F30–F39	24	2.1	17	1.4	102	1.2	512	1.3	1770	1.1	96	1.0	<.001
ICD-10 F40–F48	7	0.6	12	1.0	27	0.3	104	0.3	436	0.3	25	0.3	.037
ICD-10 F70–F79	43	3.8	23	1.9	81	0.9	296	0.7	836	0.5	65	0.7	<.001
ICD-10 F80–F89	8	0.7	7	0.6	24	0.3	97	0.2	354	0.2	25	0.3	.76
ICD-10 G80	58	5.1	16	1.3	13	0.2	15	0.0	39	0.0	NR		<.001

NR, data not reported when the number of observations is up to three, to ensure individual privacy; F00–F99, Mental, behavioural, and neurodevelopmental disorders; F20–F29, Schizophrenia, schizotypal, delusional, and other non-mood psychotic disorders; F30–F39, Mood [affective] disorders; F40–F48, Anxiety, dissociative, stress-related, somatoform, and other non-psychotic mental disorders; F70–F79, Intellectual disabilities; F80–F89, Pervasive and specific developmental disorders; G80, cerebral palsy.

a The *P* values refer to two-sided tests for differences compared with full-term birth (born at 39 + 0–41 + 6 weeks of gestation) as the reference. (Pearson’s χ2 test).

DP decisions were based on medical examinations and the certification of functional impairment relative to the requirements of the work, and granted when illness, injury, or disability reduces work ability for ≥1 year. Both fixed-term and permanent, part-time, and full-time DP decisions were included as indicators of long-term disability.

The Finnish DP system consists of two components: a residence-based DP and an earnings-related DP. Individuals who have never worked or have accrued insufficient earnings‑related DP receive the residence-based benefit, which ensures a minimum income for people with disabilities. The residence-based component applies from age 16 years onwards, while the earnings-related component applies from age 17 years onwards [24, 30–33].

### Statistical analyses

Group differences in characteristics, socio-demographic factors, and DP incidence were assessed by using Pearson’s *χ*^2^ test for categorical variables and one-way analysis of variance for continuous variables. Cox proportional-hazards regression was applied to model the time to DP. Hazard ratios (HRs) with 95% confidence intervals (CIs), adjusted according to Models 1 and 2, were obtained across the GA groups, with full-term adults as the reference. Predicted survival curves from the Cox regression were based on Model 2 adjustments. Subgroup analyses were conducted by birthweight category (SGA vs. AGA/LGA) and parental SEP.

Potential clinically relevant interactions of GA with sex, categorized birthweight SD score, being born SGA, year of birth, parental SEP, and parental educational level were tested at *P *< .01 by including a product term together with these variables in Cox regression. Interactions with a *P* value of <.01 were reported and analyses were stratified by the corresponding covariate.

Sensitivity analyses were conducted by excluding individuals (*n *= 747, 0.3%) who had never entered working life nor secondary school but had instead received residence-based DP already at age 16 years. Exclusion was based on the incidence peak of DP recipients at 16.01–16.09 years of age.

The normality of continuous variables (birthweight SD score and the age of the mother at the birth of the index person) was checked visually and statistically. The data were analysed by using IBM SPSS Statistics version 29.0 for Windows (IBM Corp., Armonk, NY, USA).

## Results

**Figure 2:**
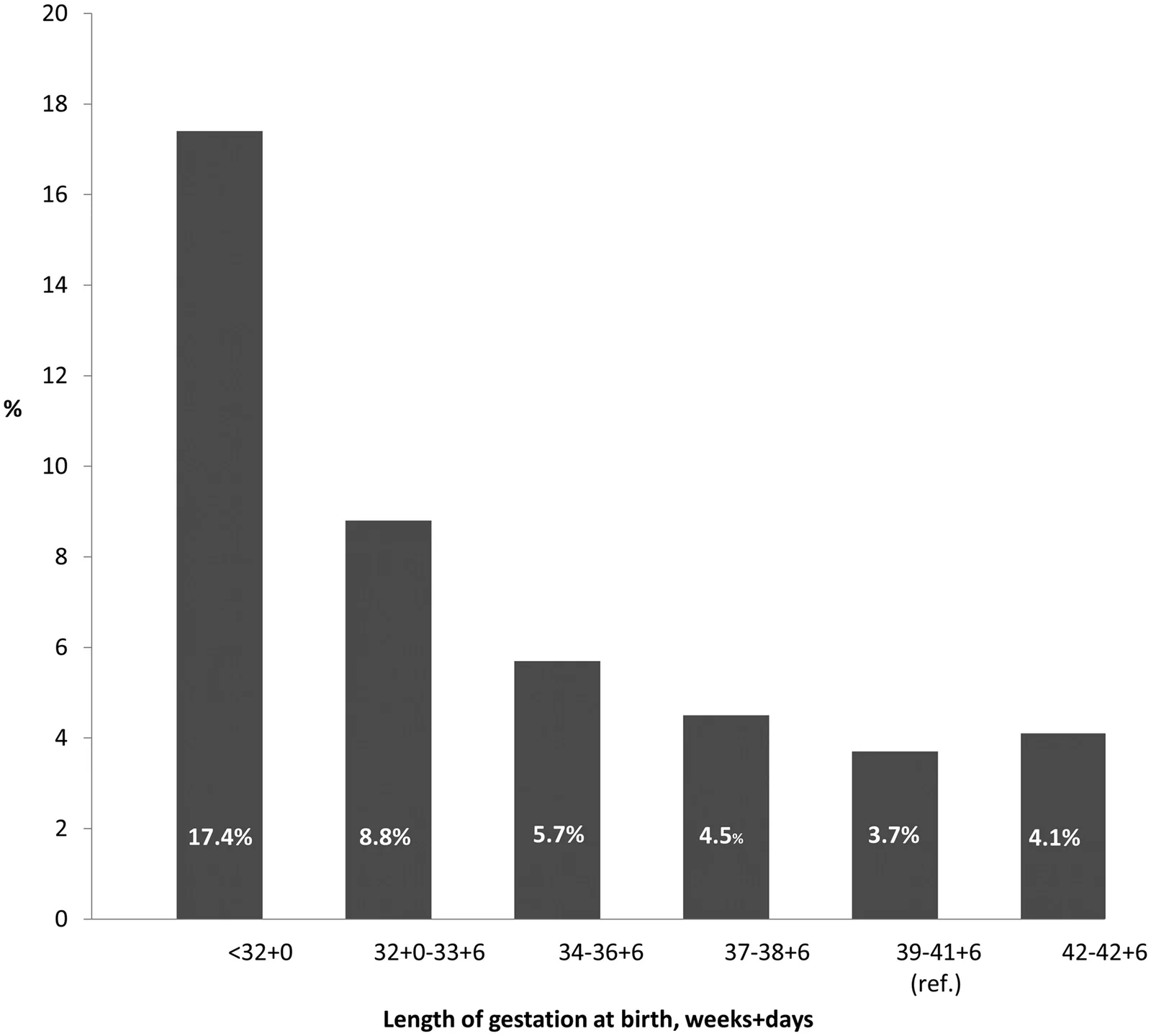
The incidence of DPs was followed among adults born in 1987–90 (*n *= 228 572) from 16 years of age until they were slightly over 30 years old, during the period from 2003 to 2020. Of the total, 9224 individuals (4.0%) received a DP at least once during the follow-up period. The proportion of DP recipients is categorized by GA groups.

**Figure 3:**
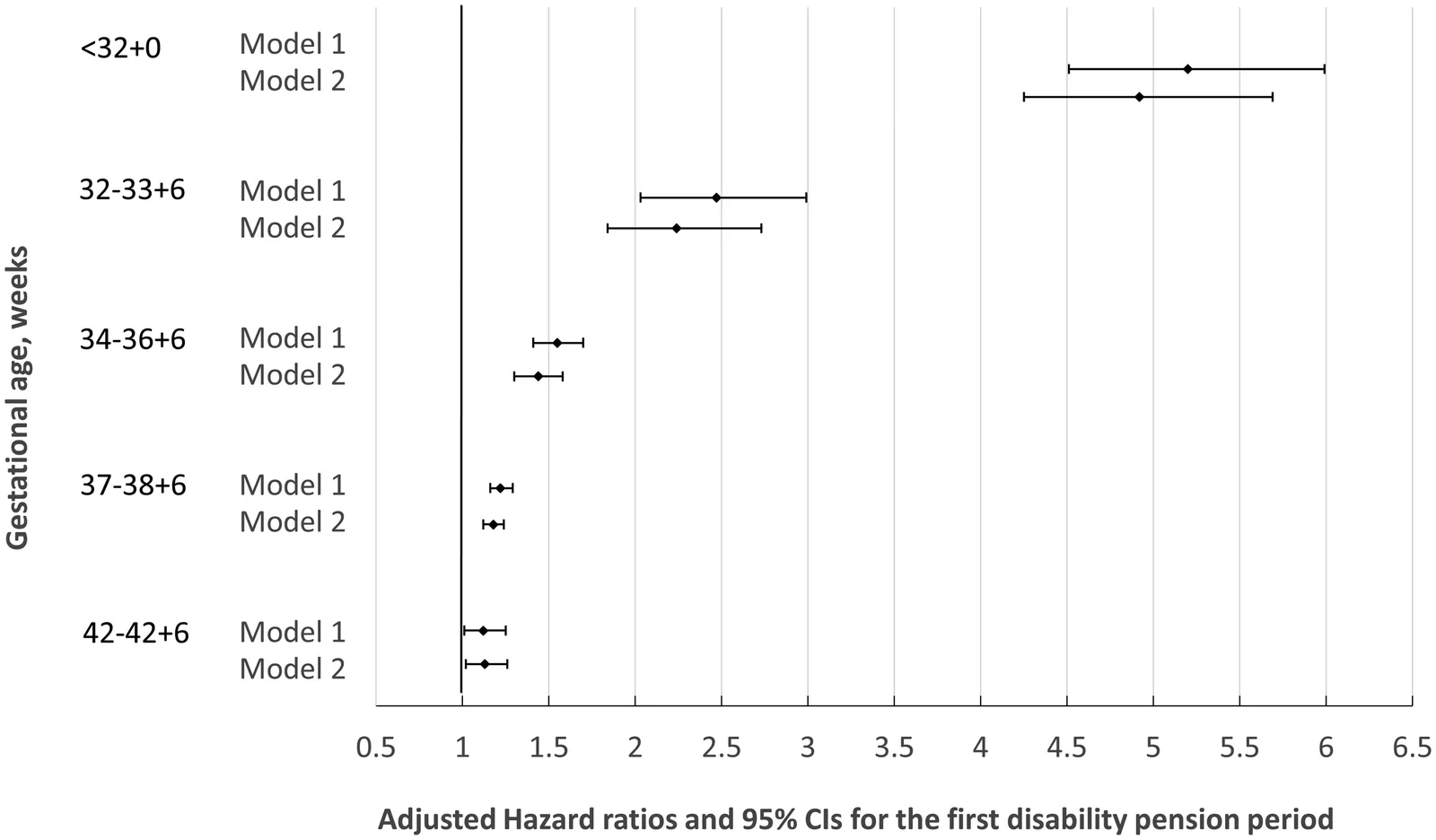
Adjusted HRs and 95% CIs for the first DP period compared with the full-term reference group (1), by GA among adults born between 1987 and 1990 (*n *= 228 572). Individuals were followed from 16 years of age until they were slightly over 30 years old, during the period from 2003 to 2020. Model 1: adjusted for sex and the year of birth. Model 2: adjusted for sex, year of birth, age of the mother at birth, parity, singleton pregnancy, maternal hypertension, maternal gestational diabetes, maternal smoking during pregnancy, birthweight SD score, parental SEP, and parental education.

**Figure 4:**
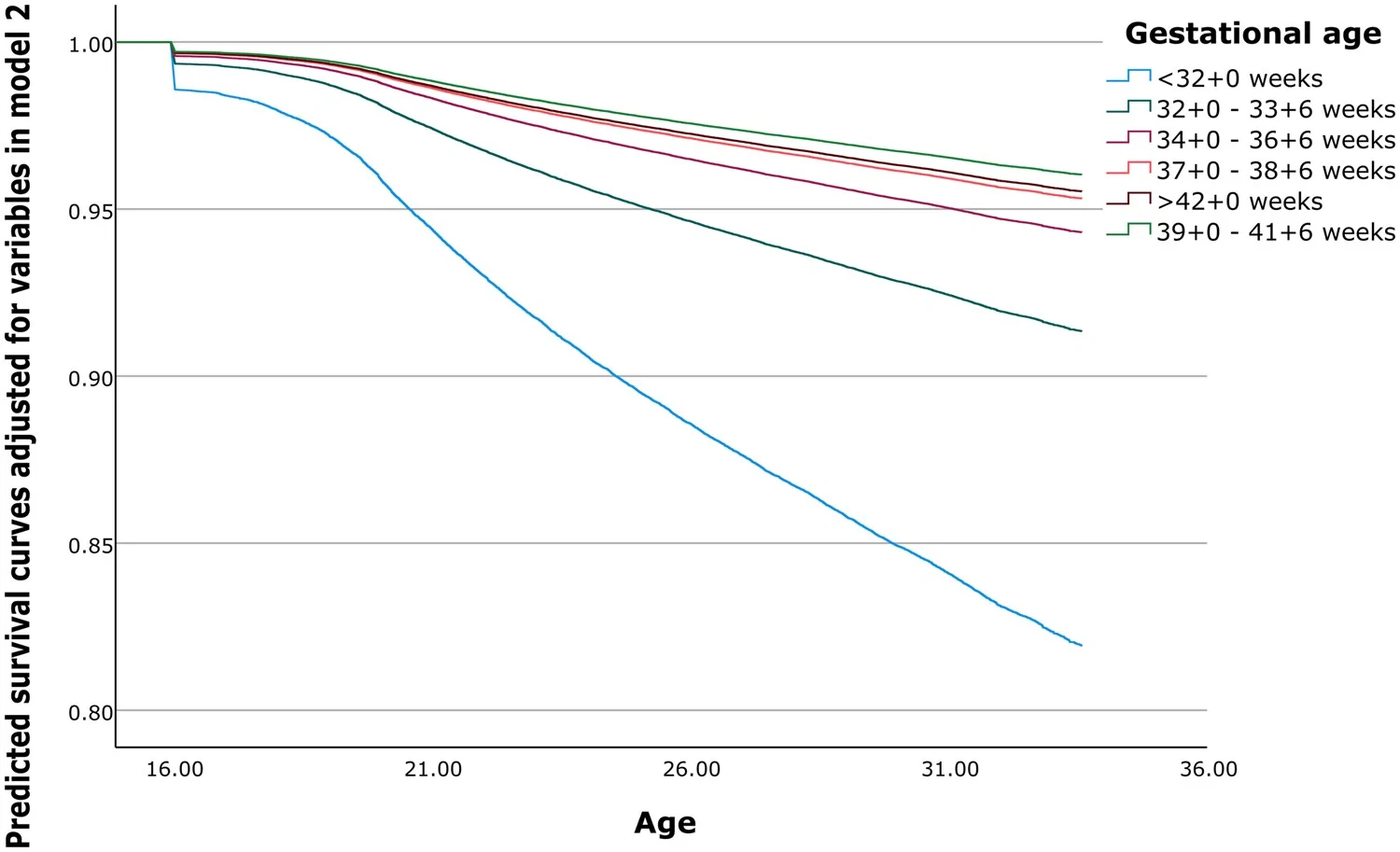
Predicted survival curves adjusted for variables in Model 2 depicting the probability of remaining free of DP over time, by GA group, among adults born from 1 January 1987 to 30 September 1990 (*n *= 228 572). Individuals were followed from 16 years of age until slightly over 30 years old, during 2003–20. Survival curves represent a male individual with categorical covariates fixed at their reference levels and continuous covariates set to their mean values.

Of the 228 572 adults, 9224 (4.0%) had received DP at least once during the follow-up at a mean age of 23.7 years (SD 4.6). The age at which individuals received DP was lower among those with lower GA (Table 4). DP incidence was 17.4%, 8.8%, 5.7%, 4.5%, 3.7%, and 4.1% by GA groups born at 23–31, 32–33, 34–36, 37–38, 39–41, and 42 weeks (Fig. 2). When adjusted for sex and year of birth (Model 1), the corresponding HRs (95% CIs) for DP compared with full-term adults were 5.20 (4.52–5.99), 2.47 (2.04–3.00), 1.55 (1.41–1.70), 1.22 (1.16–1.29), 1.0, and 1.12 (1.01–1.25), respectively (Table 2 and Fig. 3). These associations slightly weakened but remained in Model 2. Adults born very preterm (<32 weeks) had the largest decline in predicted survival (Fig. 4) based on Cox regression, followed by those born moderately preterm and late-preterm, while full-term and post-term groups showed the highest likelihood of remaining free of DP throughout the follow-up.

**Table 4:** Outcomes of DP by GA compared with the reference group (individuals born at 39 + 0–41 + 6 weeks of gestation) from rhe Finnish 1987–90 Birth Cohort study (*n *= 228 572).

Weeks of gestation (*N*)	<32 + 0 (*n *= 1138)					32 + 0–33 + 6 (*n *= 1198)				34–36 + 6 (*n *= 8548)				37–38 + 6 (*n *= 40 746)				39–41 + 6 (*n *= 167 761)			42–42 + 6 (*n *= 9182)			
Variable	*n*	%	Mean (SD)	*P* value^a^		*n*	%	Mean (SD)	*P* value^a^	*n*	%	Mean (SD)	*P* value^a^	*n*	%	Mean (SD)	*P* value^a^	*n*	%	Mean (SD)	*n*	%	Mean (SD)	*P* value^a^
DP during follow-up time	198	17.4				106	8.8			484	5.7			1834	4.5			6222	3.7		380	4.1		
Age at the first DP period (all)			21.38 (4.32)		<.001			22.31 (4.48)	.001			22.83 (4.65)	<.001			23.47 (4.64)	<.001			23.94 (4.51)			23.67 (4.61)	.777
Age at the first DP period if SGA (*n *= 484)			22.51 (4.90)		1.000			21.73 (4.30)	.988			21.71 (4.27)	.898			21.76 (4.78)	.854			22.28 (4.80)			20.94 (4.66)	.816
Age at the first DP period if AGA (*n *= 8740)			21.22 (4.22)		<.001			22.47 (4.54)	.011			20.03 (4.69)	<.001			23.60 (4.61)	.005			24.00 (4.48)			23.79 (4.57)	.9
Age at the first DP period by SEP																								
Higher white-collar			21.54 (4.26)		<.001			23.05 (4.58)	.587			22.53 (4.62)	<.001			23.86 (4.62)	.569			24.15 (4.43)			24.82 (4.64)	.425
Lower white-collar			21.26 (4.27)		<.001			21.72 (4.33)	.007			23.07 (4.89)	.025			23.43 (4.68)	.011			24.01 (4.54)			23.43 (4.62)	.451
Manual workers			20.91 (4.61)		.003			22.57 (5.06)	.797			23.01 (4.52)	.659			22.94 (4.56)	.093			23.59 (4.54)			22.66 (4.20)	.383
Other			22.12 (4.48)		.862			21.48 (2.70)	.796			22.02 (3.53)	.407			23.19 (4.68)	.998			23.36 (4.56)			22.37 (4.46)	.850
DP already at 16 years old	42	3.7				16	1.3			68	.8			171	.4			418	.2		32	.3		
DP later than already at 16 years old	156	13.7				90	7.5			416	4.9			1663	4.1			5804	3.5		348	3.8		
Age at the first DP period (all)			22.81 (3.74)		<.001			23.42 (3.93)	.063			23.94 (4.04)	.029			24.23 (4.19)	.062			24.51 (4.12)			24.38 (4.16)	.98
Age at the first DP period if SGA (*n *= 381)			24.66 (3.40)		.974			22.58 (3.95)	.528			23.13 (3.56)	.561			23.26 (4.24)	.534			24.01 (4.34)			23.38 (3.73)	.993

*P* values regarding categorical analyses from Pearson’s *χ*^2^ test for differences compared with those born at 39 + 0–41 + 6 weeks of gestation were <.05 except for ‘DP during follow-up time’.

a The *P* values refer to two-sided tests for differences compared with those born at 39 + 0–41 + 6 weeks of gestation (two-way analysis of variance).

Group ‘Other’ includes individuals not engaged in any occupation, those in military or civil service, family members without their own occupation, persons living on property income or savings, institutional inmates, or cases for which socio-economic status cannot be determined. This group also includes students, retired persons, and long-term unemployed.

Supplementary Table 1 presents the HRs for DP by GA and birthweight category (SGA vs. AGA/LGA). Among very preterm individuals, the DP risk did not differ by birthweight, whereas, in other GA groups, being SGA was linked to additional risk. Supplementary Table 2 shows that lower parental SEP was associated with a higher DP risk across all GA groups, with associations attenuated but persisting in Model 2.

In the interaction analysis, no evidence of interactions with a *P* value of <.01 was found between GA and sex (*P* for interaction = .176), categorized birthweight SD score (*P *= .174), SGA status (*P *= .338), parental SEP (*P *= .926), or parental education (*P *= .677) in relation to DP risk. The interaction analysis showed evidence of an interaction between GA and year of birth (*P *< .001) in relation to DP risk. Therefore, the DP risk was estimated by birth year (1987–90), using full-term births in 1987 as the reference (Supplementary Table 3). This analysis showed that preterm birth was consistently linked to higher DP risk across all years, although the difference was smaller in 1987. Among early-term adults, elevated risk appeared in 1988–90 and, among post-term adults, in 1989–90.

In the category of Mental, behavioural, and neurodevelopmental disorders (ICD-10 F00–F99), when adjusted for sex and year of birth (Model 1), the corresponding HRs (95% CIs) for DP compared with full-term adults were 3.64 (3.01–4.40), 2.25 (1.80–2.82), 1.40 (1.25–1.56), 1.19 (1.12–1.26), and 1.20 (1.07–1.34), respectively (Table 2). These associations slightly weakened but remained in Model 2. The most common diagnostic groups within the F00–F99 category were mood disorders (F30–F39, 35.2%), psychotic disorders (F20–F29, 25.4%), and intellectual disabilities (F70–F79, 18.8%). Adults born moderately preterm had a 2.26-fold HR for DP due to phobic anxiety disorders (F40–F48) compared with those born full-term (95% CI: 1.24–4.11).

For intellectual disabilities (F79), and after adjusting for Model 1 variables, the HRs (95% CIs) for DP increased with earlier GA, being 2.14 (1.57–2.91), 1.88 (1.25–2.85), 1.38 (1.10–1.74), 1.38 (1.10–1.74), and 1.28 (1.00–1.65) for the groups born at 23–31, 32–33, 34–36, 37–38, and 42 weeks compared with full-term group, respectively. These associations slightly weakened but remained in Model 2 for the groups born at 23–31, 32–33, and 37–38 weeks. The association was evident for the group born at 42 weeks: 1.31 (95% CI 1.02–1.66).

DP risk due to CP (G80) was higher with decreasing GA (Table 2) among preterm adults. Compared with full-term adults and adjusted for Model 2 variables, the HRs for DP strengthened from Model 1, being 72-fold among adults born very preterm (95% CI 46.8–111.3), almost 33-fold among adults born moderately preterm (95% CI 17.8–60.2), and 5-fold among adults born late-preterm (95% CI 2.66–9.53).

### Sensitivity analyses

When those who had already been granted a DP at age 16 years were excluded, 8477 of 227 825 adults (3.7%) had received DP. The HRs for DP were slightly lower but consistent with the main findings, except for post-term adults, whose DP risk was no longer evident (Supplementary Table 4). For the F00–F99 category, the proportion of the incident DP showed a slight decline, primarily attributable to a reduction in cases related to intellectual disabilities (Supplementary Table 5). Compared with the main analyses, the HR for DP due to CP was higher among adults born very and moderately preterm.

## Discussion

We found that the predicted cumulative incidence of DP increased as GA decreased. Among adults born full-term, 3.7% had received DP compared with 17% of those born very preterm, corresponding to a 5.2-fold higher hazard. Individuals born moderately or late-preterm or early-term showed intermediate incidences (4.5%–8.8%) and HRs (1.2–2.5). Adults born post-term also had a slightly elevated risk (4.1%, HR 1.1) compared with full-term adults.

Despite the extensive literature on adult outcomes of preterm birth, the DP has rarely been examined. Our results align with those of three earlier Nordic studies showing that lower GA at birth is associated with higher DP risk [15–17], despite marked differences in eligibility criteria across countries—such as minimum ages ranging from 16 to 40 years and required work capacity reductions from 25% to 75%. Moreover, two of these studies examined cohorts born decades earlier than ours, before major advances in prenatal and neonatal care. Higher use of social benefits has been reported in earlier register studies [15, 16] and in meta-analyses of studies in adults born very preterm or with very low birthweight (<1500 g) in high-resource settings [34, 35].

Across all GA groups, the leading DP diagnoses were mental, behavioural, and neurodevelopmental disorders (ICD-10 F00–F99), with DP risk increasing clearly as GA decreased. Within F00–F99, psychotic (F20–F29) and mood (F30–F39) disorders were common overall. While previous evidence links preterm birth with elevated risks of psychiatric disorder [5, 36] including non-affective psychosis, depression [18], anxiety and mood disorders [37], and attention-deficit/hyperactivity disorder [36, 38], our findings show no GA-related pattern in their contribution to DPs. Only phobic anxiety disorders (F40–F48) showed an increased DP risk and this was limited to individuals born moderately preterm.

CP was an important contributor to DPs, particularly among adults born before 34 weeks, which is expected given its origin in early brain injury and its long‑lasting effects on motor function and work capacity [39]. Additionally, preterm, early-term, and post-term births were associated with more DP cases due to intellectual disabilities (ICD-10 F70–F79), consistently with evidence showing decreasing prevalence with increasing GA [16, 40]. Elevated risk in post-term births is less often reported, though Heuvelman and colleagues [41] found such a risk in association with fetal growth restriction, including in post-term births.

No clear association was found between GA and DP risk when considering SGA status; among very preterm individuals, GA appeared more influential than SGA on DP risk. Our finding that being born to manual-worker parents increased DP risk aligns with earlier research linking parental social adversities to an increased risk of work disability in young adulthood [19, 23].

We observed that the association between GA and DP strengthened over time. Advancements in prenatal and neonatal care could partly explain this trend. While individual-level treatment data were unavailable, the use of antenatal corticosteroids increased during the study years, with 22% of infants born before 34 weeks in 1987–9 exposed [42], and some infants received surfactants in randomized trials [43]. Improved survival among the most vulnerable infants could have amplified the long-term morbidity, reinforcing the GA–DP association in later cohorts. Evolving diagnostic practices—particularly for psychiatric conditions—may have contributed to the rising DP rates, despite unchanged eligibility criteria. Labour-market fluctuations are unlikely explanations given the required prolonged sickness-allowance period. Overall, the pattern likely reflects cumulative life-course health effects and improved diagnostic recognition rather than policy or economic shifts, consistently with the increasing youth mental-health needs that exceed the current service capacity [44].

The exclusion of individuals who had never worked or attended secondary school showed similar patterns to those in the main analyses, with a slightly lower risk of DP, mainly due to fewer cases of intellectual disability (F70–F79). DP decisions for the F20–F48 and F80–F89 categories remained relatively stable, suggesting that the DP decisions for these disorders are primarily granted in adulthood.

DPs and early labour-market exit impose substantial economic burden and the number of 20- to 35‑year‑olds granted DPs has risen by ∼50% in Finland [24, 45] and 88% in Norway [46] since 2003. Proactive rehabilitation aims to prevent permanent DPs by supporting continued employment despite reduced capacity [47], with interventions tailored to functional limitations. Recognizing the link between GA and DP risk can guide targeted support, including tailored rehabilitation and work adjustments [47]. Such measures are particularly important for those born very preterm, with the highest DP risk (HR 5.2) compared with full-term peers. At the population level, even the modest risk elevations among early-term (1.2) and post-term (1.1) births remain relevant due to the large size of these groups. However, implementing targeted actions requires deeper understanding of the underlying mechanisms.

### Strengths and limitations

Key strengths include full-cohort registry coverage, a large sample size, and physician-confirmed DP follow-up into early adulthood. We accounted for a wide range of socio-economic and perinatal confounders, including parental education and SEP, across the full GA spectrum. GA was primarily determined through ultrasound [22], ensuring high accuracy [48, 49], though minor inaccuracies are possible when based on the last menstrual period. Individuals with implausible birthweight–GA combinations were excluded, to reduce potential bias. To ensure consistency with national growth standards at between 23 and 43 weeks [27] and to minimize GA misclassification, only individuals born at 42 weeks were included in the post-term group.

Finnish register data are generally accurate and reliable [21], with statistics maintained, supported by rigorous data collection, validation, and regular updates [45]. Classification error was inherently low, with a minimal likelihood of random diagnostic error—particularly for ICD-10 F00–F99 disorders—reflected by the narrow CIs. However, 101 individuals (1.0%) had multiple DP diagnoses recorded simultaneously; for these cases, the earliest diagnosis was used, which may have introduced some misclassification.

Despite the potential bias and error inherent in register data, the large, representative cohort and consistency with previous studies suggest that these factors alone are unlikely to have explained the results. Instead, genetic and shared environmental confounding may offer an alternative explanation [4]. Additionally, a range of health-related, socio-economic, and work-life events throughout the life course—beyond the scope of this study—could have influenced the outcomes. As with all observational studies, causal inferences are not possible. As with all studies on DPs, generalizability may be limited given the substantial cross-national differences in DP eligibility criteria, healthcare systems, and labour-market structures.

## Conclusions

DPs were primarily granted due to mental, behavioural, and neurodevelopmental disorders in young adulthood. Compared with full-term birth, preterm birth was associated with a 1.6- to 5.2-fold hazard of receiving DP, with a clear dose–response relationship as GA decreased. Good perinatological care is crucial for future careers. Supporting long-term workforce participation is important to reduce the societal costs of early retirement in young adulthood.

## Ethics approval

The research protocol was approved by the coordinating ethics committee of the Hospital District of Helsinki and Uusimaa and relevant register authorities. The study was conducted in accordance with the Declaration of Helsinki.

## Supplementary Material

dyag127_Supplementary_Data

## Data Availability

The de-identified individual-level data underlying this article cannot be made publicly available. Access to the sensitive register-based data was granted only to authorized members of the study groups, in accordance with the permissions obtained from the respective register holders. Researchers interested in accessing similar data may apply for data-access permissions from the Finnish Social and Health Data Permit Authority, Findata (https://findata.fi/en/).
