# Supplementary material for: Gestational age at birth and the risk of disability pension in early adulthood: a whole-population register study of people born in Finland in 1987–90
Source: Int J Epidemiol. 2026 Jul 29;55(4):dyag127. doi: 10.1093/ije/dyag127 (PMC13418203; doi:10.1093/ije/dyag127)
Supplement: dyag127_Supplementary_Data [file dyag127_supplementary_data.pdf]

Supplementary Data

Gestational age at birth and the risk of disability pension in early adulthood: a whole-population register study of people born in Finland in 1987–1990

Contents:

Table S1

Table S2

Table S3

Table S4

Table S5

Table S1. Risk of disability pension in groups appropriate for gestational age (AGA), large for gestational age (LGA) and small for gestational age (SGA) by gestational age compared with the reference group (individuals born at 39-41+6 weeks of gestation and as AGA or LGA) from The Finnish 1987-90 Birth Cohort study *(n* = 228 572).

**Weeks of gestation**	<32+0		32+0-33+6		34-36+6		37-38+6		39-41+6		42-42+6	
	HR (95% CI)	*P* value [a]	HR (95% CI)	*P* value [a]	HR (95% CI)	*P* value [a]	HR (95% CI)	*P* value [a]		*P* value [a]	HR (95% CI)	*P* value [a]
**AGA or LGA**												
Model 1	5.26 (4.52–6.12)	<0.001	2.28 (1.84–2.83)	<0.001	1.45 (1.31–1.60)	<0.001	1.20 (1.13–1.26)	<0.001	reference		1.12 (1.01–1.25)	0.034
Model 2	5.06 (4.34–5.90)	<0.001	2.16 (1.74–2.69)	<0.001	1.39 (1.26–1.54)	<0.001	1.17 (1.11–1.24)	<0.001	reference		1.13 (1.02–1.26)	0.022
**SGA**												
Model 1	5.48 (3.67–8.18)	<0.001	4.01 (2.66–6.04)	<0.001	2.96 (2.35–3.71)	<0.001	2.42 (2.03–2.88)	<0.001	1.83 (1.60-2.10)	<0.001	2.02 1.22-3.35)	0.007
Model 2	5.25 (3.51–7.86)	<0.001	3.62 (2.39–5.47)	<0.001	2.68 (2.13–3.38)	<0.001	2.11 (1.77–2.52)	<0.001	1.65 (1.44-1.89)	<0.001	1.90 1.14-3.15)	0.013

Abbreviations: AGA, Appropriate for gestational age; CI, Confidence interval; HR, Hazard ratio; LGA, Large for gestational age; SGA, Small for gestational age

Model 1: Sex, Year of birth. Model 2: Sex, Year of birth, Age of the mother at birth, Parity (at least 1 partus before this pregnancy) (Yes vs. no), Singleton pregnancy (Yes vs. no), Maternal hypertension (Yes vs. no), Maternal gestational diabetes (Yes vs. no), Maternal smoking during pregnancy, less than 10 cigarrettes a day (Yes vs. no), Maternal smoking during pregnancy, more than 10 cigarrettes a day (Yes vs. no), Maternal smoking during pregnancy, information missing (Yes vs. no), Parental highest ever attained socioeconomic position: Lower white-collar (Yes vs. no), Manual workers (Yes vs. no), Other (Yes vs. no; includes individuals not engaged in any occupation, those in military or civil service, family members without their own occupation, persons living on property income or savings, institutional inmates, students, retired persons, long-term unemployed, or cases where socioeconomic status cannot be determined) Missing information (Yes vs. no), Parental educational level, highest ever attained: Basic or unknown, Secondary, less than tertiary, and tertiary or more.

[a] The *P* values concern the adjusted hazard ratios (HRs) with 95% confidence intervals (CIs) estimated using Cox regression, with those born as AGA or LGA and at 39+0 - 41+6 weeks of gestation as the reference.

Table S2. Risk of disability pension between parental sosioeconomic categories by gestational age compared with the reference group (individuals born at 39-41+6 weeks of gestation to higher white-collar family) from The Finnish 1987-90 Birth Cohort study *(n* = 228 572).

		<32+0 weeks of gestation		32+0-33+6 weeks of gestation		34-36+6 weeks of gestation		37-38+6 weeks of gestation		39-41+6 weeks of gestation		42-42+6 weeks of gestation	
		HR (95% CI	*P* value [a]	HR (95% CI	*P* value [a]	HR (95% CI	*P* value [a]	HR (95% CI	*P* value [a]	HR (95% CI	*P* value [a]	HR (95% CI	*P* value [a]
**Parental socio-economic position**	**Higher white-collar**												
	Model 1	5.53 (4.33–7.06)	<0.001	2.46 (1.75–3.47)	<0.001	1.39 (1.17–1.65)	<0.001	1.22 (1.11–1.33)	<0.001	reference		1.04 (0.87–1.25)	0.671
	Model 2	5.39 (4.21–6.90)	<0.001	2.35 (1.66–3.32)	<0.001	1.35 (1.14–1.61)	0.001	1.20 (1.10–1.32)	<0.001	reference		1.04 (0.87–1.25)	0.659
	**Lower white-collar**												
	Model 1	6.63 (5.33–8.24)	<0.001	2.99 (2.04–4.06)	<0.001	1.94 (1.68–2.24)	<0.001	1.54 (1.42–1.68)	<0.001	1.46 (1.24–1.71)	<0.001	1.29 (1.22–1.37)	<0.001
	Model 2	6.31 (5.05–7.88)	<0.001	2.73 (2.01–3.72)	<0.001	1.81 (1.56–2.10)	<0.001	1.48 (1.35–1.62)	<0.001	1.26 (1.18–1.34)	<0.001	1.42 (1.21–1.68)	<0.001
	**Manual workers**												
	Model 1	7.70 (5.48–10.80)	<0.001	4.51 (3.02–6.75)	<0.001	3.15 (2.60–3.81)	<0.001	2.21 (1.98–2.47)	<0.001	1.85 (1.72–1.99)	<0.001	2.26 (1.79–2.86)	<0.001
	Model 2	6.94 (4.93–9.76)	<0.001	3.83 (2.56–5.75)	<0.001	2.77 (2.28–3.37)	<0.001	1.99 (1.77–2.24)	<0.001	1.71 (1.58–1.86)	<0.001	2.08 (1.64–2.64)	<0.001
	**Other [b]**												
	Model 1	10.03 (5.93–16.96)	<0.001	4.66 (2.22–9.80)	<0.001	2.77 (1.97–3.88)	<0.001	2.29 (1.90–2.76)	<0.001	1.80 (1.60–2.01)	<0.001	2.59 (1.71–3.95)	<0.001
	Model 2	8.33 (4.91–14.12)	<0.001	3.61 (1.71–7.59)	0.001	2.41 (1.71–3.39)	<0.001	2.06 (1.71–2.49)	<0.001	1.68 (1.49–1.90)	<0.001	2.42 (1.59–3.69)	<0.001

Abbreviations: CI, Confidence interval; HR, Hazard ratio.

Model 1: Sex, Year of birth. Model 2: Sex, Year of birth, Age of the mother at birth, Parity (at least 1 partus before this pregnancy) (Yes vs. no), Singleton pregnancy (Yes vs. no), Maternal hypertension (Yes vs. no), Maternal gestational diabetes (Yes vs. no), Maternal smoking during pregnancy, less than 10 cigarrettes a day (Yes vs. no), Maternal smoking during pregnancy, more than 10 cigarrettes a day (Yes vs. no), Maternal smoking during pregnancy, information missing (Yes vs. no), Birth weight SD score, Parental educational level, highest ever attained: Basic or unknown, Secondary, less than tertiary, and tertiary or more

[a] The *P* values concern the adjusted hazard ratios (HRs) with 95% confidence intervals (CIs) estimated using Cox regression, with those born at 39-41+6 weeks of gestation to higher white-collar family as the reference.

[b] Group "Other" includes individuals not engaged in any occupation, including those in military or civil service, family members without their own occupation, persons living on property income or savings, institutional inmates, or cases where socioeconomic status cannot be determined. This group also includes students, retired persons, and long-term unemployed.

Table S3. Risk of disability pension between birth years by gestational age compared with the reference group (individuals born at 39-41+6 weeks of gestation during the year 1987) from The Finnish 1987-90 Birth Cohort study (*n* = 228 572).

**Weeks of gestation**		**<32+0 , *n*=1138**				**32+0-33+6, *n*=1198**				**34-36+6, *n*=8548**				**37-38+6, *n*=40 746**				**39-41+6, *n*=167 761**				**42-42+6, *n*=9182**			
Year	M	*n*	%	HR (95% CI)	*P* value [a]	*n*	%	HR (95% CI)	*P* value [a]	*n*	%	HR (95% CI)	*P* value [a]	*n*	%	HR (95% CI)	*P* value [a]	*n*	%	HR (95% CI)	*P* value [a]	*n*	%	HR (95% CI)	*P* value [a]
**1987**	1	49	4.3	2.7 (2.0–3.6)	<0.001	20	1.7	1.7 (1.1–2.6)	0.025	123	1.44	1.4 (1.1–1.6)	0.001	481	1.2	1.1 (1.0–1.2)	0.251	1697	1.0	reference		93	1.01	1.1 (0.9–1.3)	0.499
	2			2.6 (2.0–3.5)	<0.001			1.5 (1.0–2.4)	0.059			1.3 (1.0–1.5)	0.017			1.0 (0.9–1.2)	0.469			reference				1.1 (0.9–1.4)	0.367
**1988**	1	58	5.1	7.0 (5.4–9.1)	<0.001	35	2.9	3.2 (2.3–4.5)	<0.001	140	1.64	1.8 (1.5–2.1)	<0.001	513	1.3	1.3 (1.2–1.4)	0.001	1668	1.0	1.1 (1.0–1.2)	0.013	90	0.98	1.1 (0.9–1.3)	0.167
	2			6.9 (5.3–8.9)	<0.001			2.9 (2.1–4.1)	<0.001			1.7 (1.4–2.0)	<0.001			1.3 (1.2–1.4)	0.001			1.1 (1.0–1.2)	0.007			1.2 (1.0–1.5)	0.126
**1989**	1	48	4.2	5.4 (4.1–7.2)	<0.001	27	2.3	2.5 (1.7–3.7)	<0.001	126	1.47	1.7 (1.4–2.1)	<0.001	464	1.1	1.4 (1.2–1.5)	<0.001	1627	1.0	1.2 (1.1–1.2)	<0.001	102	1.11	1.2 (1.0–1.5)	0.035
	2			5.1 (3.8–6.8)	<0.001			2.2 (1.5–3.2)	<0.001			1.6 (1.4–3.0)	<0.001			1.3 (1.2–1.5)	<0.001			1.2 (1.1–1.3)	<0.001			1.3 (1.0–1.5)	0.024
**1990 (Jan-Sep)**	1	43	3.8	6.5 (4.8–8.7)	<0.001	24	2.0	3.0 (2.0–4.5)	<0.001	95	1.11	1.7 (1.4–2.1)	0.001	376	0.9	1.5 (1.3–1.6)	0.001	1230	0.7	1.1 (1.1–1.2)	0.002	95	1.03	1.5 (1.2–1.8)	<0.001
	2			6.1 (4.5–8.2)	<0.001			2.6 (1.8–4.0)	<0.001			1.7 (1.3–2.0)	<0.001			1.4 (1.3–1.6)	<0.001			1.2 (1.1–1.2)	0.001			1.5 (1.2–1.8)	<0.001

Abbreviations: CI, Confidence interval; HR, Hazard ratio; M, Model.

Model 1: Sex Model 2: Sex, Age of the mother at birth, Parity (at least 1 partus before this pregnancy) (Yes vs. no), Singleton pregnancy (Yes vs. no), Maternal hypertension (Yes vs. no), Maternal gestational diabetes (Yes vs. no), Maternal smoking during pregnancy, less than 10 cigarrettes a day (Yes vs. no), Maternal smoking during pregnancy, more than 10 cigarrettes a day (Yes vs. no), Maternal smoking during pregnancy, information missing (Yes vs. no), Birth weight SD score, Parental highest ever attained socioeconomic position: Lower white-collar (Yes vs. no), Manual workers (Yes vs. no), Other (Yes vs. no; includes individuals not engaged in any occupation, those in military or civil service, family members without their own occupation, persons living on property income or savings, institutional inmates, students, retired persons, long-term unemployed, or cases where socioeconomic status cannot be determined), Missing information (Yes vs. no), Parental educational level, highest ever attained: Basic or unknown, Secondary, less than tertiary, and tertiary or more.

[a] The *P* values concern the adjusted hazard ratios (HRs) with 95% confidence intervals (CIs) estimated using Cox regression, with those born at 39-41+6 weeks of gestation and during the year 1987 as the reference.

Table S4. Risk of disability pension and related key diagnoses by gestational age compared with the reference group (individuals born at 39+0 - 41+6 weeks of gestation) among those who received the disability pension later than already at 16 years of age from The Finnish 1987-90 Birth Cohort study (*n* = 227 825).

**Weeks of gestation, *n***		**<32+0 , *n*=1138**				**32+0-33+6, *n*=1198**				**34-36+6, *n*=8548**				**37-38+6, *n*=40 746**				**39-41+6, *n*=167 761**		**42-42+6, *n*=9182**			
	**M**	***n***	**%**	**HR (95% CI)**	***P* value [a]**	***n***	**%**	**HR (95% CI)**	***P* value [a]**	***n***	**%**	**HR (95% CI)**	***P* value [a]**	***n***	**%**	**HR (95% CI)**	***P* value [a]**	***n***	**%**	***n***	**%**	**HR (95% CI)**	***P* value [a]**
**All disability pension causes together**	1	156	14.2	4.4 (3.8–5.2)	<0.001	90	7.6	2.3 (1.8–2.8)	<0.001	416	4.91	1.4 (1.3–1.6)	<0.001	1663	4.1	1.2 (1.1–1.3)	<0.001	5804	3.5	348	3.80	1.1 (1.0–1.2)	0.070
	2			4.2 (3.6–4.9)	<0.001			2.1 (1.7–2.5)	<0.001			1.3 (1.2–1.5)	<0.001			1.2 (1.1–1.2)	<0.001					1.1 (1.0–1.2)	0.058
**F00-F99**	1	91	8.3	3.3 (2.7–4.0)	<0.001	67	5.7	2.1 (1.7–2.7)	<0.001	308	3.63	1.3 (1.2–1.5)	<0.001	1310	3.2	1.2 (1.1–1.3)	<0.001	4625	2.8	291	3.18	1.2 (1.0–1.3)	0.014
	2			3.0 (2.5–3.7)	<0.001			1.9 (1.5–2.4)	<0.001			1.2 (1.1–1.4)	0.001			1.2 (1.1–1.2)	<0.001					1.2 (1.0–1.3)	0.012
F20-F29	1	19	1.7	0.8 (0.5–1.3)	0.43	15	1.3	0.9 (0.6–1.6)	0.796	83	0.98	1.0 (0.8–1.3)	0.972	324	0.8	0.9 (0.8–1.0)	0.071	1285	0.8	88	0.96	1.2 (0.9–1.4)	0.195
	2			0.8 (0.5–1.2)	0.27			0.9 (0.5–1.5)	0.866			1.0 (0.8–1.2)	0.776			0.9 (0.8–1.0)	0.033					1.2 (0.9–1.4)	0.185
F30-F39	1	22	2.0	0.8 (0.5–1.2)	0.19	17	1.4	0.8 (0.5–1.3)	0.296	101	1.19	0.9 (0.8–1.1)	0.44	511	1.3	1.1 (1.0–1.2)	0.325	1767	1.1	96	1.05	0.9 (0.7–1.1)	0.274
	2			0.7 (0.5–1.1)	0.13			0.8 (0.5–1.2)	0.273			0.9 (0.7–1.1)	0.322			1.0 (0.9–1.2)	0.465					0.9 (0.7–1.1)	0.240
F40-F48	1	6	0.5	0.8 (0.4–1.8)	0.56	11	0.9	2.1 (1.1–3.7)	0.019	27	0.32	1.0 (0.7–1.5)	0.934	104	0.3	0.9 (0.7–1.1)	0.176	436	0.3	25	0.27	1.00 (0.6–1.4)	0.820

	2			0.8 (0.4–1.8)	0.56			2.1 (1.1–3.9)	0.02			1.0 (0.7–1.5)	0.97			0.9 (0.7–1.1)	0.183					0.9 (0.6–1.4)	0.754
F70-F79	1	28	2.6	2.2 (1.5–3.2)	<0.001	15	1.3	1.9 (1.1–3.1)	0.018	51	0.60	1.3 (1.0–1.7)	0.108	210	0.5	1.3 (1.1–1.5)	0.003	592	0.4	41	0.45	1.2 (0.9–1.6)	0.332
	2			2.0 (1.4–3.0)	<0.001			1.6 (0.9–2.7)	0.098			1.1 (0.8–1.4)	0.729			1.2 (1.0–1.4)	0.021					1.2 (0.9–1.6)	0.278
F80-F89	Model 1	8	0.7	1.4 (0.7–2.7)	0.39	7	0.6	1.7 (0.8–3.7)	0.148	21	0.25	1.0 (0.6–1.6)	0.995	91	0.2	1.0 (0.8–1.3)	0.941	321	0.2	21	0.23	1.1 (0.7–1.7)	0.682
	Model 2			1.3 (0.6–2.6)	0.55			1.5 (0.7–3.2)	0.352			0.9 (0.6–1.5)	0.76			1.0 (0.8–1.3)	0.932					1.1 (0.7–1.7)	0.748
**G80**		43	3.9	82 (50–134)	<0.001	12	1.0	34 (17–68)	<0.001	8	0.09	4.6 (2.1–10.1)	<0.001	8	0.0	1.1 (0.5–2.5)	0.787	26	0.0			NR	
				94 (56–158)	<0.001			40 (19–83)	<0.001			4.9 (2.2–10.9)	<0.001			1.2 (0.5–2.6)	0.73						

Abbreviations: CI, Confidence interval; HR, Hazard ratio; M, Model; NR, Data not reported when the number of observations is ≤3 to ensure individual privacy; F00-F99, Mental, behavioral and neurodevelopmental disorders; F20-F29, Schizophrenia, schizotypal, delusional, and other non-mood psychotic disorders; F30-F39, Mood [affective] disorders; F40-F48, Anxiety, dissociative, stress-related, somatoform and other nonpsychotic mental disorders; F70-F79, Intellectual Disabilities; F80-F89, Pervasive and specific developmental disorders; G80, Cerebral palsy.

Model 1: Sex, Year of birth. Model 2: Sex, Year of birth, Age of the mother at birth, Parity (at least 1 partus before this pregnancy) (Yes vs. no), Singleton pregnancy (Yes vs. no), Maternal hypertension (Yes vs. no), Maternal gestational diabetes (Yes vs. no), Maternal smoking during pregnancy, less than 10 cigarrettes a day (Yes vs. no), Maternal smoking during pregnancy, more than 10 cigarrettes a day (Yes vs. no), Maternal smoking during pregnancy, information missing (Yes vs. no), Birth weight SD score, Parental highest ever attained socioeconomic position: Lower white-collar (Yes vs. no), Manual workers (Yes vs. no), Other (Yes vs. no; includes individuals not engaged in any occupation, those in military or civil service, family members without their own occupation, persons living on property income or savings, institutional inmates, students, retired persons, long-term unemployed, or cases where socioeconomic status cannot be determined)), Missing information (Yes vs. no), Parental educational level, highest ever attained: Basic or unknown, Secondary, less than tertiary, and tertiary or more.

[a] The *P* values concern the adjusted hazard ratios (HRs) with 95% confidence intervals (CIs) estimated using Cox regression, with those born at 39+0 - 41+6 weeks of gestation as the reference.

Table S5. Disability pension frequencies of key diagnoses by gestational age compared with the reference group (individuals born at 39+0 - 41+6 weeks of gestation) among those who received the disability pension later than already at 16 years of age from The Finnish 1987-90 Birth Cohort study (*n* = 227 825).

**Weeks of gestation, *n***	**<32+0 , *n*=1138**		**32+0-33+6, *n*=1198**		**34-36+6, *n*=8548**		**37-38+6, *n*=40 746**		**39-41+6, *n*=167 761**		**42-42+6, *n*=9182**		
**ICD-10 group**	*n*	%	*n*	%	*n*	%	*n*	%	*n*	%	*n*	%	*P* value [a]
ICD-10 F00-F99	91	8.3	67	5.7	308	3.6	1310	3.2	4625	2.8	291	3.2	<0.001
ICD-10 F20-F29	19	1.7	15	1.3	83	1.0	324	0.8	1285	0.8	88	1.0	0.003
ICD-10 F30-F39	22	2.0	17	1.4	101	1.2	511	1.3	1767	1.1	96	1.0	<0.001
ICD-10 F40-F48	6	0.5	11	0.9	27	0.3	104	0.3	436	0.3	25	0.3	0.093
ICD-10 F70-F79	28	2.6	15	1.3	51	0.6	210	0.5	592	0.4	41	0.4	0.001
ICD-10 F80-F89	8	0.7	7	0.6	21	0.2	91	0.2	321	0.2	21	0.2	0.937
ICD-10 G80	43	3.9	12	1.0	8	0.1	8	0.0	26	0.0	NR		0.001

Abbreviations: NR, Data not reported when the number of observations is ≤3 to ensure individual privacy; F00-F99, Mental, behavioral and neurodevelopmental disorders; F20-F29, Schizophrenia, schizotypal, delusional, and other non-mood psychotic disorders; F30-F39, Mood [affective] disorders; F40-F48, Anxiety, dissociative, stress-related, somatoform and other nonpsychotic mental disorders; F70-F79, Intellectual Disabilities; F80-F89, Pervasive and specific developmental disorders; G80, Cerebral palsy.

[a] The *P* values refer to two-sided test for differences compared with full-term birth (born at 39+0 - 41+6 weeks of gestation) as the reference. (Pearson's χ^2 test)
